# Current and potential role of grain legumes on protein and micronutrient adequacy of the diet of rural Ghanaian infants and young children: using linear programming

**DOI:** 10.1186/s12937-019-0435-5

**Published:** 2019-02-21

**Authors:** Ilse de Jager, Karin J. Borgonjen-van den Berg, Ken E. Giller, Inge D. Brouwer

**Affiliations:** 1 Division of Human Nutrition and Plant Production Systems group, Wageningen University, Wageningen, The Netherlands; 2 Division of Human Nutrition and Health, Wageningen University, Wageningen, The Netherlands; 3 Plant Production Systems group, Wageningen University, Wageningen, The Netherlands

**Keywords:** Grain legumes, protein, essential amino acids, micronutrients, nutrient adequacy, optimised diets, infants and young children

## Abstract

**Background:**

Grain legumes are appreciated for their contribution to dietary protein and micronutrient intake in addition to their benefits in providing income and replenishing soil fertility. They offer potential benefits in developing countries where future food demand is increasing and both undernutrition and overweight co-exist. We studied the current and potential role of grain legumes on protein, both quantity and quality, and micronutrient adequacy in the diet of rural Ghanaian infants and young children.

**Methods:**

Energy and nutrient (including amino acids) intakes of breastfed children of 6-8 months (*n*=97), 9-11 months (*n*=97), 12-23 months (*n*=114), and non-breastfed children of 12-23 months (*n*=29) from Karaga district in Northern Ghana were assessed using a repeated quantitative multi-pass 24-hour recall method. Food-based dietary guidelines that cover nutrient adequacy within the constraints of local current dietary patterns were designed using the linear programming software Optifood (version 4.0.9, Optifood©). Optifood was also used to evaluate whether additional legumes would further improve nutrient adequacy.

**Results:**

We found that 60% of the children currently consumed legumes with an average portion size of 20 g per day (cooked) contributing more than 10% of their total protein, folate, iron and niacin intake. The final sets of food-based recommendations included legumes and provided adequate protein and essential amino acids but insufficient calcium, iron, niacin and/or zinc among breastfed children and insufficient calcium, vitamin C, vitamin B12 and vitamin A among non-breastfed children. The sets of food-based recommendations combined with extra legumes on top of the current dietary pattern improved adequacy of calcium, iron, niacin and zinc but only reached sufficient amounts for calcium among breastfed children of 6-8 months old.

**Conclusions:**

Although legumes are often said to be the ‘meat of the poor’ and current grain legume consumption among rural children contribute to protein intake, the main nutritional benefit of increased legume consumption is improvement of micronutrient adequacy. Besides food-based recommendations, other interventions are needed such as food-based approaches and/or fortification or supplementation strategies to improve micronutrient adequacy of infants and young children in rural Ghana.

**Trial registration:**

Noguchi Memorial Institute for Medical Research Institutional Review Board (NMIMR-IRB CPN 087/13-14).

**Electronic supplementary material:**

The online version of this article (10.1186/s12937-019-0435-5) contains supplementary material, which is available to authorized users.

## Background

Grain legumes^1^ can play a significant role in food systems to address future global food security, environmental sustainability and nutritional needs [1–3]. Driven by climate change, urbanization, income growth and population increase, food systems are transforming rapidly and often fail to provide sufficient, diverse, nutritious and safe food for all [2, 4]. Grain legumes are appreciated for their contribution to dietary protein and micronutrient intake in addition to their benefits in providing cash income for smallholders and replenishing soil nutrients. Legumes have the unique ability to fix atmospheric nitrogen in the soil, reduce fertilizer requirements and increase yield in subsequent crops [5]. Compared with maize, one of the most commonly consumed staple globally, legumes are better sources of protein (20 to 30 percent) and are richer in the key micronutrients folate, niacin, thiamine, calcium, iron and zinc, although nutrient concentration vary considerably between grain legumes, varieties and locations [6–8]. Human nutrient uptake from legume consumption greatly depends on the bioavailability of nutrients [9, 10]. In addition, being a good source of essential amino acids (EAAs), especially of lysine, grain legumes are complementary to most staple foods, improving the protein quality of the diet [6–8, 11, 12]. Grain legumes offer potential benefits in developing countries where future food demand is increasing [4] and undernutrition and overweight co-exist [13].

The current productivity of most legumes is lowest in developing countries, especially in sub-Saharan Africa [14, 15]. Overall the availability of legumes together with dairy, meat, fruits, nuts and seeds has declined in sub-Saharan Africa while the availability of grains less-dense in protein and micronutrients has increased [16]. Protein intake is often estimated to be inadequate in sub-Saharan Africa, both in terms of quantity and quality [17]. Nevertheless, these estimations were not based on estimated dietary intakes and therefore the evidence is weak. More than 30% of children are stunted in Africa, the only continent where the number of stunted children has risen from 2000 to 2016 [18]. Several cross-sectional studies suggest that dietary intake of essential amino acids (EAAs) are insufficient in stunted children, especially that of lysine which is the most limiting EAA in cereal based diets [19–21]. A recent randomised controlled trial among Ghanaian infants from age 6 to 18 months was conducted and preliminary results showed a dose-response effect of receiving a protein quality and micronutrient-improved complementary food supplement on their growth at 18 months of age [22, 23]. Based on food balance sheet data, thet prevalence of inadequate micronutrient intake decreased in sub-Saharan Africa from 1990 due to increased total energy supplies and/or dietary micronutrient density [16]. Deficiencies in micronutrients such as iron, iodine, vitamin A, folate and zinc affect more than 2 billion people worldwide; again with the highest prevalence in sub-Saharan Africa. The greatest concern is for infants and young children (IYC) as micronutrient deficiencies impair their mental and physical development resulting in life-long irreversible disadvantages [24, 25].

Increasing the availability and consumption of legumes in sub-Saharan Africa has potential to close the protein and micronutrient gaps. Suri DJ, Tano-Debrah K and Ghosh SA [26] found that traditional cereal–legume blends made from locally available ingredients in Ghana had improved protein quality and micronutrients compared with a traditional Ghanaian maize-based complementary food (koko) but still did not meet quality protein and micronutrient recommendations**.** However, optimisation of these food blends, including added fat, amino acids, and micronutrients, may result in meeting nutrient requirements [26]. Yet evidence on actual consumption and nutrient contribution of legumes is limited. Available data show large variation between regions and age groups. For example, only 44% of rural IYC in southern Ethiopia consumed legumes and/or nuts which contributed less than 4% of their total protein intake [27]. By contrast more than 90% of school-age children in northern Ghana consumed legumes and/or nuts although no information was available on the contribution to protein or micronutrient intake [28]. These are the only studies we can find that have investigated the current contribution of legumes to EAAs intakes of IYC in developing countries Optimisation studies developing food-based recommendations (FBRs) based on current dietary patterns of IYC, show that combinations of local foods including legumes improve but do not provide adequate amounts of all nutrients [29–32]. However, none of these studies included adequacy of EAAs in their analyses, nor did they test whether inclusion of a further increase of legume consumption would potentially be able to reach protein and nutrient adequacy.

We collected quantitative dietary intake data among IYC in rural Northern Ghana and used it to: (a) identify grain legumes consumption and contribution to nutrients in the current diet, (b) identify a set of food-based recommendations that will improve nutrient adequacy within the constraints of local current dietary patterns, and (c) evaluate whether including extra grain legumes on top of what is normally consumed would reduce the number of problem nutrients which are present in relatively high concentrations in grain legumes (protein, EAAs, calcium, folate, iron, niacin and zinc).

## Methods

### Study area

The study was carried out in Karaga sub-district in the Northern Region of Ghana. Cultivation and consumption of grain legumes, especially cowpea (*Vigna unguiculata* (L.) Walp) and groundnut (*Arachis hypogaea* L.), is common in this region. Karaga sub-district was selected because of high food insecurity and malnutrition. About 32% of children below 5 years old are stunted and 9.4% are wasted [33].

### Subjects

Infants and young children between 6-23 months are the primary target of this study divided into the four following groups: breastfed infants between 6-8 months (6-8 BF), breastfed infants between 9-11 months (9-11 BF), breastfed young children between 12-23 months (12-23 BF) and non-breastfed young children between 12-23 months (12-23 NBF). A census was conducted in Karaga sub-district between May-June 2014 to identify all households with children of 6-23 months and collect information on their sex, date of birth (from verifiable documents (health record, weighing card, birth certificate) or estimated based on traditional calendar), breastfeeding status and geographical location by GPS coordinates. A list of all households with children of 6-23 months constituted the sampling frame divided into four sub-frames, corresponding to the four specific groups according to age and breastfeeding state: 6-8 BF, 9-11 BF, 12-23 BF and 12-23 NBF. A random order list was developed for each sub-frame and the first 100 children on this list were selected except in case there were less than 100 children in a group.

Eligibility was defined by the age of the child falling between 6-23 months using the day before the start of data collection as the reference date (30 June 2014). For the breastfed group, eligibility was also defined as receiving both breastfeeding and complementary feeding. Eligibility for the study was cross-checked in the field prior to the start of data collection and ineligible children were randomly replaced with other eligible children in the same community or a nearby community. A sample size of approximately 100 for each of the four groups was chosen based on estimated population mean food serving sizes for commonly-consumed foods in the study area to be within 10% (95% CI), assuming an SD of 50% of the mean serving sizes in the age group and allowing for a 5% rate of attrition. This sample size is comparable to those previously used in studies with linear programming techniques in the literature [34]. One child per household was selected. In case two or more children in the household qualified for inclusion, one was chosen randomly. Communities of selected children were clustered into three geographic areas: north, central and south. Each cluster was then randomly assigned to a time slot of data collection. A random sample of food vendors within the selected study communities and major markets within the study area were also interviewed to determine prices of foods identified during collection of dietary data. Food price data were used for estimation of quantities of reported foods consumed, as well as to calculate the daily diet costs of each child which in turn was used as a criterion for the final selection of feasible FBRs.

### Data collection and analysis

Data was collected in Ghana in July 2014 by trained enumerators who had a first degree in nutrition and who spoke the local language. Trained supervisors with previous experience in dietary assessment and who spoke the local language, observed part of the interviews and back-checked survey forms of all interviews. In case of inconsistencies, households were revisited.

### Anthropometry

Weight and length of children were measured in duplicate following WHO guidelines [35] using an electronic scale (UNIscale: Seca GmbH, Hamburg, Germany) and an UNICEF wooden three piece measuring board with a sliding foot piece. The scale was calibrated daily. Anthropometric indices were calculated based on the WHO Child Growth Standards [36] using the WHO SPSS syntax. Children were classified as stunted and wasted if their height-for-age and weight-for-height Z-score was less than minus two, respectively. Children were classified as overweight if their BMI-for-age Z-score was more than two.

### Dietary intake assessment

Dietary intakes of the children were assessed using a quantitative multi-pass 24-hour recall method [37] with all days evenly distributed over the week. A second recall was carried out for 20% of the children on a non-consecutive day to permit adjustment for day-to-day variation of nutrient intakes. Data was collected in a time period of 3 weeks. Primary caretakers were asked to recall all the foods and drinks consumed in and outside the home by their child during the preceding day and to describe ingredients and cooking methods of any mixed dishes. To assess the amounts of the foods and ingredients, similar foods were weighed to the nearest 2 g using a Soehnle electronic kitchen scale (Plateau Art 65086, Germany). Scales were randomly assigned to the interviewers and calibrated daily. When the actual food was not available in the household, amounts were estimated (in order of priority) as their monetary value equivalents (price paid at the market and converted to quantity that was bought using the food price data collected), compared the weight of other foods (e.g. amount of sugar estimated with weight of same volume of corn flour), in volumes, as their general sizes (small, medium or large) using pictures or in household units (such as a spoon or bowl). Conversion factors were applied to convert these units into grams of the foods consumed to be able to assess nutrient intake. The total volume of each (mixed) dish cooked at the respondents’ household and the volume of this dish specifically consumed by the child were measured to determine the proportion of the dish consumed by the child. This proportion was multiplied by the total amount of ingredients used in the preparation of the dish to determine the amount of ingredients consumed. Standard recipes were generated to estimate the weight of ingredients consumed from mixed dishes eaten outside the home by averaging three recipes of different vendors in the local area. For each food consumed by the children, food price data was also collected from three different food sellers in the study area to calculate the price per edible 100 g portion of all foods.

### Habitual dietary intake

Energy and nutrient intakes were calculated using nutrient calculation system Compl-eat^TM^ (version 1.0, Wageningen University), including: energy, carbohydrates, fat, protein, EAAs (histidine, isoleucine, leucine, lysine, threonine, tryptophan, valine, aromatic amino acids (AAA, include phenylalanine and tyrosine) and sulphur-containing amino acids (SAA, include methionine and cystine); calcium, vitamin C, thiamine, riboflavin, niacin, vitamin B6, folate, vitamin B12, vitamin A, iron, and zinc. Energy and nutrient intake calculations were based on a food composition table (FCT) specifically created for this study using the West African FCT as primary source [7] complemented with data from FCTs from, in order of priority based on date of publication and location with similar dietary pattern, Mali FCT [38], the United States Department of Agriculture database [6] and the Ghana FCT [39]. EAA values in gram per 100 gram protein were derived from the recent elaborate Indian FCT [11] that uses validated methods to measure AAs content in foods, and applied to the protein content derived from the FCTs listed above. If a specific food was not included in the Indian FCT, a similar food from the same food group and with similar protein content was selected. Several processed food items were not included in the Indian FCT; for these items the proportion of ingredients was used to derive the EAAs content. The nutrient composition of breast milk was taken from the WHO as the vitamin A content was reported to be more representative of developing countries [40]. Energy content of breast milk was assumed to be 65 kcal per 100 g. EAA values in breastmilk were taken from a recent systematic review by Zhang Z, Adelman AS, Rai D, Boettcher J and Lőnnerdal B [41] on amino acid profiles in human milk including a few studies from Africa. Where appropriate, yield [7] and nutrient retention factors [6, 42] were applied to account for nutrient losses during food preparation. If only the raw food items were included in the Indian FCT these were used assuming the different preparation methods do not affect the relative proportion of EAAs contents. The Atwater general factors for carbohydrate, protein and fat and the recommended metabolisable energy for dietary fibre in ordinary diets (2 kcal or 8.4 kJ/g) were used in calculating energy [43]. Total vitamin A was calculated as retinol activity equivalent (RAE) by the sum of retinol and 1/12 β-carotene [7]. Energy and nutrient intake were analysed using statistical software package IBM SPSS (version 23). Normality of distributions was tested visually using QQ plots. Non-normal nutrient intake data were log transformed, resulting in normal distributions. To generate usual intakes, nutrient intakes were adjusted for day-to-day variation using the National Research Council adjustment method [44, 45]. For breastfed children, intake of breastmilk was not measured directly and therefore we assumed average intakes based on estimated energy intakes from breastmilk for populations in low income countries [40, 46]. The total nutrient intake for breastfed children were computed by their adjusted nutrient intakes plus the nutrient intake from the assumed average breastmilk intakes [40]. Energy and nutrient intakes are reported as median (25^th^, 75^th^ percentile) of the distribution of intakes.

The percentage of children for all four groups (6-8 BF, 9-11 BF, 12-23 BF and 12-23 NBF) with energy and macronutrient intakes below their daily requirements (see Additional file 1 for values used) and with micronutrient intakes below EARs when available (see Additional file 2) were also determined. The daily median intake and contribution of grain legumes to energy and nutrient intakes (in mean % ± SD) was determined for all four groups. In addition, we divided our target population of children 6-23 months into two groups: children who did and children who did not consume grain legumes and tested the differences in total energy and nutrient intakes between these two groups with the Mann-Whitney U test. Two-sided *P*-value <0.05 was regarded as statistically significant.

### Optimising dietary intake

The linear programming software Optifood (version 4.0.9, Optifood©) was used to design population-specific FBRs [30, 32, 47]. The model parameters were defined per target group and generated using Microsoft® Excel 2010, IBM SPSS (version 23) and Microsoft® Access 2010, based on the 24-hour recall data of the first day. The parameters included: a list of non-condiment foods consumed by ≥ 5% of the target children or ≥ 5 children for the non-breastfed children and excluding fortified foods, for each selected food the price per 100 g of edible food (to determine price of modelled diets) and for each selected food the median serving size for all children who had consumed it. The minimum and maximum number of servings per week for each (sub)food groups were defined as the 5^th^ and 95^th^ percentile distributions of serving counts. The minimum and maximum frequencies per individual food within a (sub)food group was estimated based on percentage of children consuming that food. For energy and nutrient contents of the foods, the FCT table specifically developed for this study was also used in Optifood. All modelled diets had to meet the energy requirements for the specific target group, estimated using reference mean body weight and the FAO/WHO/UNU algorithm for estimating energy requirements [48]. Thirteen nutrients were considered in the Optifood analysis: total fat, total protein, calcium, vitamin C, thiamine, riboflavin, niacin, vitamin B6, folate, vitamin B12, vitamin A, iron and zinc. EAAs were included in the Optifood analysis as well if at least 10% among one of the target groups had a daily intake below one of their EAAs daily requirements. For fat the requirements were based on the acceptable macronutrient distribution range (ADMR) of 30% of daily energy requirements [49]; for protein based on average reference mean body weight for age group and algorithm for estimating protein requirement (g/kg), safe intakes [50]; for EAAs based on daily total protein requirements and algorithms for each EAAs requirements (mg/g protein) using safe intakes [50]; and for other micronutrients RNIs were used from FAO/WHO [51], except for zinc the RNI from the International Zinc Nutrition Consultative Group’s (iZiNCG) reflecting low bioavailability of unrefined cereal-based diets [52] was used. Considering the low dietary haem iron with high phytate and fibre in the plant foods commonly consumed by our target groups, 5% bioavailability was assumed for iron [51].

Module 1-3 were used in the Optifood analyses for all target groups. Module 1 was run to check that model parameters generated diets that are feasible for the target population. Module 1 generates 19 different diets including poor, middle and nutrient rich diets and shows the energy range of these diets and a high range is preferred as this shows flexibility of the model. Module 2 was run to identify the best optimised diet that met or come as close as possible to meeting nutrient needs of the target population but is constrained by the minimum and maximum number of servings per week. The objective function was to minimize the deviation of the current diet while reaching the nutrient goals. The best optimised diet was used to select FBRs to test in Module 3, including the food groups with weekly servings above zero and individual foods contributing at least 5% to the intake of one of the nutrients. In Module 3, two diets were modelled for each nutrient of which one maximized nutrients selecting the most nutrient dense foods within each food group to verify the highest possible nutrient intake (the maximised diet) and one minimized nutrients selecting the lowest nutrient dense foods to verify the lowest possible nutrient intake (the minimised diet). The objective function was to respectively minimize and maximize each nutrient. First, module 3 was run without FBR constraints to identify problem nutrients of which the RNI cannot be met by any combination of currently consumed local foods (defined as below 100% RNI in the maximised diets). As Optifood software has a maximum of 14 nutrients that can be considered, nutrients not considered as problem nutrients in all of the four target groups (>100% RNI in maximised diets) were no longer included in the linear programming analyses and replaced by the EAAs that meet the inclusion criteria described above. Second, individual and combined FBRs were tested to identify sets of FBRs that covered >70% of the RNI in the minimized diet for most nutrients and total costs below the 75^th^ percentile of daily diet cost. Nutrient intakes above 70% of RNI in the minimized diet were classified as adequate, for most nutrients this represents at least the EAR, and it allows for comparison with other studies [30, 32, 34]. For each target group, the set of recommendations that achieved >70% of the RNI in the minimized diet for most nutrients but below the 75^th^ percentile of daily diet cost was selected (see Additional file 3 for the specific criteria used for each group). Third, extra grain legumes were incorporated in this final set of selected FBRs and tested in Module 3 to determine if they improved problem nutrient adequacy. Grain legumes were added when they were consumed by all four groups with a median portion size of above 3 g and when they contained larger concentrations of at least one of the problem nutrients of a target group compared with the staple food maize. The minimum and maximum number of servings per week for each grain legume were set at 7 assuming that the addition of one extra serving of a specific grain legume per day was feasible within the energy constraints. When 7 servings did exceed the energy constraints, the maximal number of servings that were possible within the energy constraints were added. The median portion size for ‘new’ legumes (consumed by <5% of children in all four target groups) incorporated in final FBRs was calculated as the average of the median portion size per group assuming to be a more feasible portion size than the median portion size of each group being consumed by less than 5% of the target children. Adding a combination of different legumes to the final set of FBRs, was only carried out when it did not exceed the energy constraints. Again, for each target group the set of recommendations that achieved >70% of the RNI in the maximised diet for most nutrients but below the 75^th^ percentile of daily diet cost was selected.

## Results

### Subject characteristics

**Fig. 1:**
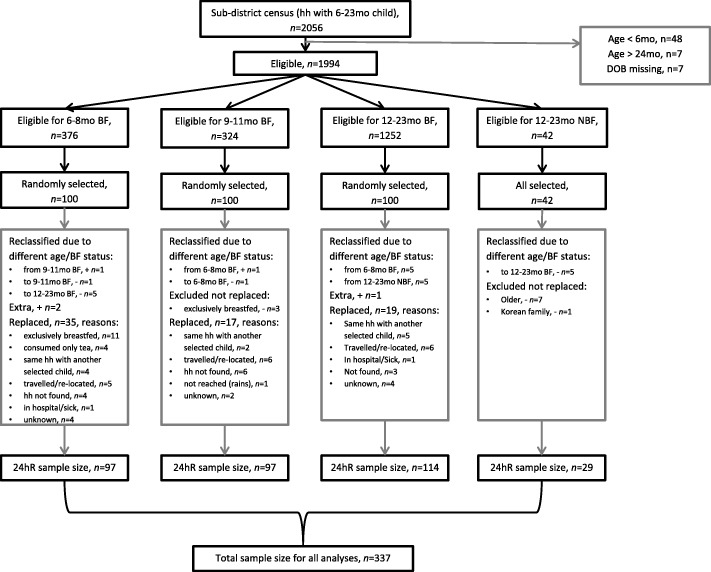
Flow chart of sample selection. hh = household. n=sample size. 6-8 BF = breastfed children of 6-8 months, 9-11 BF = breastfed children of 9-11 months, 12-23 BF = breastfed children of 12-23 months, 12-23 NBF = non-breastfed children of 12-23 months. Reclassified=from other age group to this group (different age or breastfeeding status during 24hour recall than census). 24hR=24hour recall

In total 337 children were included in the study: 97 children 6-8 BF, 97 children 9-11 BF, 114 children 12-23 BF and 29 children 12-23 NBF. If eligibility criteria were not met, children were reclassified to another group or replaced in the field (Fig. 1). In the study area, 42 children of 12-23 months did not receive breastmilk of which 29 children were included as when cross-checked in the field, seven were older than 23 months, five did receive breastmilk and one was from a Korean family with different dietary habits compared with target children.

Children were on average 8, 11, 17 and 21 months old, respectively among children of 6-8 BF, 9-11 BF, 12-23 BF and 12-23 NBF. About 50 % of children were girls in all groups except in group of children 12-23 NBF where 38 % of children were girls. Among children below 12 months about 30 % were stunted, while among the older children above 12 months about 55 % were stunted. Among all children, about 14 % were wasted. One child 12-23 NBF was overweight (Table 1).

**Table 1:** Nutritional status of children 6 to 23 months old in Karaga sub-district, Northern region, Ghana^a^

	6-8 BF	9-11 BF	12-23 BF	12-23 NBF
Characteristics	*n*=97^b^	n=97^c^	*n*=114	*n*=29
Age, months	7.9 ± 0.9	10.8 ± 1.0	17.1 ± 3.2	20.9 ± 3.3
Sex, girls, % (n)	52.6 (51)	52.6 (51)	50.0 (57)	37.9 (11)
Height for age, z score	-1.2 ± 1.1	-1.6 ± 1.2	-2.2 ± 1.3	-1.9 ± 2.1
Children being stunted % (n)	26.8 (26)	31.9 (31)	53.5 (61)	55.2 (16)
Weight for height, z score	-1.0 ± 1.0	-1.0 ± 1.1	-1.0 ± 0.9	-0.8 ± 1.3
Children being wasted % (n)	14.4 (14)	13.4 (13)	13.2 (15)	13.7 (4)
Body-mass-index for age, z score	-1.1 ± 1.0	-0.9 ± 1.1	-0.7 ± 0.9	-0.4 ± 1.3
Children begin overweight, % (n)	0 (0)	0 (0)	0 (0)	3.4 (1)

6-8 BF = breastfed children of 6-8 months, 9-11 BF = breastfed children of 9-11 months, 12-23 BF = breastfed children of 12-23 months, 12-23 NBF = non-breastfed children of 12-23 months

^a^Values are mean ± standard deviation unless stated otherwise

^b^*n*=96, missing anthropometric measurements information for 1 child

^c^*n*=96, missing date of birth and anthropometric measurements information for 1 child

### Habitual dietary intake

Data analysis included 337 first dietary recalls and 66 second recalls (20%). In all four groups, with average breastmilk intakes assumed, about 50% of children had an energy intake below their daily requirement (also reflected in the high prevalence of wasted children) while nearly all children had sufficient fat or protein intakes. All children had essential amino acid intakes above their requirements, except for isoleucine, lysine and/or AAA intakes. Micronutrient intakes were generally low, for children above 12 months for almost all nutrients 20% or more children had intakes below their daily requirements. For more than 60% of children above 12 months calcium, folate, and vitamin B12 were below their requirements, and in addition for the non-breastfed children also iron, vitamin A and vitamin C. For children below 12 months, 90% had iron and zinc intakes below their requirements and for folate 50% of 6-8 months old children and 35% of 9-11 months old children (for other nutrients no EARs were available) (Table 2).

**Table 2:** Daily nutrient intake of children 6 to 23 months old, recommended daily requirements and percent below daily requirements, assuming average breastmilk intake and reference weights

	6-8 BF				9-11 BF				12-23 BF				12-23 NBF			
	*n*=97				*n*=97				*n*=114				*n*=29			
	Median intake [25^th^, 75^th^ percentile], recommended daily requirements^a^, % below daily requirements^b^															
Energy (kcal)	601	[525, 721]	614	**53, 28** ^c^	717	[587, 887]	695	**49, 24** ^**c**^	897	[725, 1168]	886	**47, 22** ^**c**^	983	[773, 1284]	886	38, 24^c^
Fat (g)	28	[27, 31]	20.5	0	29	[27, 36]	23.2	0	33	[28, 40]	29.5	31	22	[13, 29]	29.5	**76**
Protein (g)	11	[10, 15]	9.1	14	14	[11, 20]	10.3	12	21	[15, 28]	11.1	11	27	[20, 35]	11.1	0
*Histidine (mg)*	*308*	*[260, 383]*	*182*	*0*	*369*	*[300, 520]*	*196*	*0*	*545*	*[388, 727]*	*199*	*1*	*695*	*[551, 918]*	*199*	*0*
^1^ *Isoleucine (mg)*	*442*	*[442, 442]*	*291*	*0*	*413*	*[412, 414]*	*324*	*0*	*369*	*[368, 370]*	*343*	*0*	*3*	*[2, 5]*	*343*	***100***
*Leucine (mg)*	*1296*	*[1069, 1606]*	*601*	*0*	*1467*	*[1243, 2020]*	*664*	*0*	*2182*	*[1630, 2875]*	*697*	*0*	*2669*	*[1921, 3461]*	*697*	*0*
*Lysine (mg)*	*690*	*[628, 791]*	*519*	*0*	*762*	*[659, 947]*	*561*	*1*	*991*	*[787, 1227]*	*575*	*5*	*991*	*[693, 1302]*	*575*	*17*
*SAA (mg)*	*441*	*[379, 551]*	*255*	*0*	*522*	*[414, 714]*	*278*	*0*	*756*	*[572, 1010]*	*288*	*1*	*992*	*[653, 1311]*	*288*	*0*
*AAA (mg)*	*673*	*[673, 673]*	*473*	*0*	*628*	*[628, 629]*	*504*	*0*	*561*	*[561, 562]*	*509*	*0*	*3*	*[2, 5]*	*509*	***100***
*Threonine (mg)*	*518*	*[460, 627]*	*282*	*0*	*590*	*[500, 785]*	*298*	*0*	*827*	*[631, 1049]*	*299*	*0*	*923*	*[697, 1265]*	*299*	*0*
*Tryptophan (mg)*	*180*	*[192, 227]*	*77*	*0*	*212*	*[185, 258]*	*82*	*0*	*257*	*[212, 313]*	*82*	*0*	*226*	*[154, 302]*	*82*	*0*
*Valine (mg)*	*680*	*[579, 861]*	*391*	*0*	*782*	*[650, 1094]*	*437*	*0*	*1174*	*[891, 1520]*	*465*	*2*	*1377*	*[1063, 1950]*	*465*	*0*
Calcium (mg)	202	[193, 239]	400	n/a	208	[187, 258]	400	n/a	227	[199, 280]	500	**95**	158	[95, 305]	500	**86**
Folate (μg DFE)	65	[61, 75]	80	**51**	72	[63, 91]	80	35	92	[75, 119]	150	**76**	81	[59, 139]	150	**66**
Iron (mg)	2	[1, 3]	18.6	**91**	3	[2, 5]	18.6	**91**	5	[3, 8]	11.6	**17**	9	[7, 13]	0	**66**
Niacin (mg)	1.9	[1.6, 3.1]	4	n/a	2.7	[1.9, 4.0]	4	n/a	4.9	[3.2, 6.8]	6	**47**	6.8	[4.7, 10.3]	6	21
Riboflavin (mg)	0.3	[0.3, 0.4]	0.4	n/a	0.3	[0.3, 0.4]	0.4	n/a	0.4	[0.3, 0.6]	0.5	41	0.5	[0.3, 0.7]	0.5	38
Thiamine (mg)	0.3	[0.3, 0.4]	0.3	n/a	0.4	[0.3, 0.5]	0.3	n/a	0.6	[0.4, 0.9]	0.5	18	0.9	[0.7, 1.1]	0.5	0
Vitamin A (μg RAE)	331	[331, 344]	400	n/a	322	[309, 354]	400	n/a	300	[287, 335]	400	24	60	[28, 142]	400	**86**
Vitamin B_6_ (mg)	0.2	[0.2, 0.4]	0.3	n/a	0.3	[0.2, 0.5]	0.3	n/a	0.6	[0.4, 0.9]	0.5	25	1.0	[0.7, 1.4]	0.5	3
Vitamin B_12_ (μg)	0.7	[0.7, 0.7]	0.7	n/a	0.6	[0.6, 0.7]	0.7	n/a	0.6	[0.6, 0.6]	0.9	**84**	0.1	[0.0, 0.3]	0.9	**86**
Vitamin C (mg)	27	[27, 30]	30	n/a	28	[25, 32]	30	n/a	28	[25, 32]	30	23	12	[5, 18]	30	**90**
Zinc (mg)	1.6	[1.3, 2.2]	5	**91**	2.1	[1.6, 3.0]	5	**91**	3.2	[2.3, 4.5]	3	13	4.4	[3.3, 6.7]	0	0

6-8 BF = breastfed children of 6-8 months, 9-11 BF = breastfed children of 9-11 months, 12-23 BF = breastfed children of 12-23 months, 12-23 NBF = non-breastfed children of 12-23 months

SAA = sulphur-containing amino acids (methionine and cystine); AAA = aromatic amino acids (phenylalanine and tyrosine). **Bold** values = percentages higher than 45

^a^Recommended daily requirements (used in Optifood analyses): for energy based on average reference body weight for age group and algorithm for estimating energy requirements (Kcal/kg) (FAO, 2004); for fat based on the acceptable macronutrient distribution range (ADMR) of 30% of daily energy requirements (FAO, 2010); for protein based on average reference body weight for age group and algorithm for estimating protein requirement (g/kg) using safe intakes (FAO, 2007); for essential amino acids based on daily total protein requirements and algorithms for each essential amino acid requirements (mg/g protein) using safe intakes (WHO, 2007); for micronutrients RNIs from FAO/WHO (2004)

^b^Percent below daily requirements: for energy and macronutrients requirements used as described above; for micronutrients based on EARs calculated from RNIs (FAO/WHO 2004), using conversion factors (Allen, et al., 2006) except for zinc based on EAR from iZiNCG (2004) assuming unrefined cereal-based diets and for iron based on RNI from FAO/WHO (2004) assuming 5% bioavailability, except for calcium, niacin, riboflavin, thiamine, vitamin A, vitamin B6, vitamin B12 and Vitamin C for children 6 to 11 months for whom EARs are not available *(see* Additional files 1 and 2 *for overview of exact values used)*

^c^Percent of children below daily requirements based on actual weight

Overall, 17, 30, 33 and 22 non-condiment foods were consumed, respectively, by more than 5 % of 6-8 BF, 9-11 BF and 12-23 BF children and by more than 5 children of 12-23 NBF (See Additional file 4). Sugar, maize flour and anchovies were the foods most commonly consumed foods by all four target groups. Serving sizes in the diet varied between 1 g/d for different fish foods, dried soybean *(Glycine max* (L.) Merril)*,* dried groundnut and dried okro powder to 123 g/d for maize flour and 126 g/d for watermelon. All vegetables were consumed in portion sizes below 30 g/d. Median portion sizes consumed of legumes, nuts and seeds were ranging from 4 to 25 g/day (except for dried soybeans and groundnuts shelled). The estimated 75^th^ percentile of daily diet costs ranges from 0.39 Ghanaian Cedi’s (GH₵) for children 6-8 BF to 2.29 GH₵ for children 12-23 NBF (See Additional file 3). Additional files 4 show minimum and maximum frequencies of individual foods consumed per target group, ranging between 0 and 7 times per week. Additional file 5 shows the minimum and maximum frequencies for sub food groups and food groups consumed, ranging between 0 and 35 times per week.

Cowpea whole, groundnut paste and soybean flour were consumed by all four target groups with median portion sizes above 3 g. Compared with maize, these grain legumes are relatively high in protein, EAAs (especially soybean), iron**,** zinc, folate and calcium (Table 3). Groundnuts are also relatively high in niacin. Median total daily legumes intake ranged from 5.2g among 6-8 BF children to 35.2g among 12-23 NBF children. Median daily intake from cowpea was the highest (31 ± 43 g/d, *n*=45) while groundnut was consumed by most children (10 ± 16 g/d, *n*=186). Soybean was consumed only by 27 children with median portion sizes of 7 ± 9.5 g/d. Among children of above 12 months, legumes currently contributed more than 10% to total protein, EAAs (especially soybean to lysine and tryptophan, and cowpea to all EAAs), folate (especially cowpea), iron (especially cowpea) and niacin (especially groundnuts) intake (Table 3) and among the non-breastfed children also to energy, fat, calcium, thiamine and zinc intake. In the diet of children below 12 months, the contribution of legumes to energy or any nutrient was below 10% with the largest contribution to protein, iron, niacin and/or zinc. Among all children, 60% consumed legumes and their total energy and most nutrient intakes were better compared with children who did not, except for isoleucine and AAA intakes (Table 3). The same comparison separately for each age group and for breastfed and non-breastfed showed similar results.

**Table 3:** Grain legumes: nutrient composition per 100 gram edible portion compared with staple food maize^a^, percent contribution of all legumes in current diet to nutrient intakes per target group, and comparison of nutrient intakes of children not consuming legumes and children consuming legumes

	Soybean flour	Groundnut paste	Cowpea, whole	Maize	6-8 BF *n=97*		9-11 BF *n=97*		12-23 BF *n=114*		12-23 NBF *n=29*		Children, not consumed legumes *n=135*		Children, consumed legumes *n=202*	
	content per 100 gram edible portion				legume contribution: mean % ± standard deviation								median intake [25^th^, 75^th^ percentile]			
Daily legume intake^b^ (g)					5.2 ± 22.3		5.5 ± 11.3		17.0 ± 30.3		35.2 ± 36.9		-		19.8 ± 31.3	
Energy (kcal)	414	578	320	349	2.0	± 7.4	2.7	± 5.2	5.5	± 7.2	**11.7**	± 10.2	596	[521, 688]	893	[726, 1142]*
Fat (g)	15.9	45.9	1.5	4.1	1.5	± 5.1	2.8	± 5.6	7.6	± 9.8	**23.0**	± 23.9	27	[26, 29]	33	[28, 39]*
Protein (g)	**34.7**	**22.4**	**19.2**	9.2	4.1	± 12.7	5.6	± 10.4	**11.0**	± 12.8	**20.3**	± 16.4	11	[10, 14]	21	[15, 28]*
Histidine (g)	***0.8***	***0.5***	***0.63***	*0.25*	*3.8*	*± 12.1*	*5.3*	*± 9.9*	***10.7***	*± 12.7*	***20.4***	*± 17.0*	*300*	*[262, 364]*	*539*	[404, 726]*
Isoleucine (g)	***1.6***	***1.1***	***0.84***	*0.34*	*3.7*	*± 11.4*	*4.9*	*± 8.8*	***10.6***	*± 12.0*	***22.0***	*± 17.2*	*441*	*[412, 442]*	***370***	[369, 414]*
Leucine (g)	***2.9***	*1.4*	*1.54*	*1.12*	*3.1*	*± 10.4*	*4.1*	*± 8.0*	*7.6*	*± 9.8*	***14.8***	*± 12.6*	*1264*	*[1068, 1575]*	*2113*	[1068, 1575]*
Lysine (g)	***2.7***	***0.8***	***1.36***	*0.24*	*4.0*	*± 12.7*	*4.8*	*± 9.2*	***11.3***	*± 13.7*	***25.9***	*± 21.6*	*674*	*[622, 754]*	*942*	[779, 1192]*
SAA (g)	***1.1***	***0.6***	*0.40*	*0.34*	*3.0*	*± 10.0*	*3.7*	*± 7.1*	*7.1*	*± 8.8*	***13.8***	*± 11.9*	*426*	*[375, 525]*	*745*	[565, 1030]*
AAA (g)	***2.7***	***2.1***	***1.71***	*0.81*	*3.8*	*± 11.9*	*5.4*	*± 9.8*	***11.2***	*± 12.8*	***21.4***	*± 17.4*	*672*	*[628, 673]*	***563***	[561, 629]*
Threonine (g)	***1.2***	***0.6***	***0.79***	*0.29*	*3.3*	*± 10.9*	*4.2*	*± 8.0*	*9.0*	*± 11.3*	***19.0***	*± 16.4*	*513*	*[452, 588]*	*804*	[624, 1044]*
Tryptophan (g)	***0.6***	***0.2***	***0.17***	*0.06*	*3.1*	*± 9.9*	*3.5*	*± 6.5*	*8.9*	*± 10.6*	***22.4***	*± 18.6*	*187*	*[174, 210]*	*251*	[212, 303]*
Valine (g)	***1.7***	***0.9***	***1.02***	*0.50*	*3.4*	*± 11.2*	*4.4*	*± 8.5*	*8.8*	*± 10.6*	***17.5***	*± 14.6*	*665*	*[578, 806]*	*1151*	[865, 1511]*
Calcium (mg)	**185**	**61**	**61**	19	1.9	± 7.7	1.3	± 2.6	4.3	± 7.7	**12.0**	± 15.2	194	[187, 223]	230	[200, 273]*
Folate (μg DFE)	**133**	**88**	**143**	18.2	4.1	± 13.8	5.1	± 9.6	**12.3**	± 15.6	**24.9**	± 22.3	63	[60, 69]	91	[76, 121]*
Iron (mg)	**5.2**	**3.9**	**6.8**	2.9	5.2	± 17.3	7.1	± 16.1	**9.5**	± 14.2	**12.3**	± 13.3	2.0	[1.5, 3.0]	5.6	[3.5, 8.5]*
Niacin (mg)	1.2	**14.7**	1.8	1.9	3.8	± 12.6	7.2	± 12.7	**14.3**	± 15.9	**20.8**	± 16.6	1.9	[1.5, 2.4]	4.7	[3.2, 6.7]*
Zinc (mg)	**4.3**	**2.0**	**4.1**	1.55	4.5	± 15.2	5.5	± 11.6	9.0	± 13.1	**15.4**	± 14.5	1.6	[1.4, 2.1]	3.2	[2.3, 4.5]*

6-8 BF = breastfed children of 6-8 months, 9-11 BF = breastfed children of 9-11 months, 12-23 BF = breastfed children of 12-23 months, 12-23 NBF = non-breastfed children of 12-23 months

SAA = sulphur-containing amino acids (methionine and cystine); AAA = aromatic amino acids (phenylalanine and tyrosine)

**Bold** values = more than 1.5 times higher nutrient content compared with maize; average above 10% contribution to nutrient intake; and nutrient intakes lower among children consuming legumes compared to children not consuming legumes

*P <0.05 (comparing nutrient intakes of children who did and did not consume legumes). The same comparison separately for each age group and for breastfed and non-breastfed showed similar results

^a^Values are from West African Food Composition Table (2012) with retention factors applied: for soybean and cowpea ‘legumes 2-2,5 hours boiled and water used’, for groundnut ‘nuts boiled’, and for maize ‘cereal boiled’ (USDA). For essential amino acids content, the proportion of amino acids of protein from comparable foods from Indian Food Composition Tables (2017) together with protein content from WAFCT were used

^b^Daily legume intake in median ± SD, for all children

### Optimised dietary intake

In Module 2 in the best optimised diets for all four groups, groundnut paste and cowpea both contributed more than 5% to the intake of at least four nutrients (See Additional file 6). Breastmilk contributed more than 5% of intake to the highest number of nutrients (13 and 14 nutrients) in all three groups with breastfed children, while in non-breastfed group this was maize flour, cowpea and groundnut paste (11 nutrients) (See Additional file 6). In Module 3 for all four groups, the maximised diets for each specific nutrient without recommendations covered the RNI for most nutrients. Among children below 12 months problem nutrients were calcium, iron and zinc, among 12-23 BF children calcium and iron, and among 12-23 NBF children calcium, vitamin B12, vitamin A and vitamin C (Tables 4 and See Additional file 7). Neither thiamine or vitamin B6 were problem nutrients in all four groups (>100% RNI in the maximised diet) and were therefore excluded for further Optifood analyses while the EAAs isoleucine, AAA and lysine were added (more than 10% children were below daily requirements) but were not identified as problem nutrients. The final sets of FBRs selected did not cover calcium, iron, niacin and/or zinc above 70% of RNI in the minimised diets for breastfed children and calcium, vitamin C, vitamin B12 and vitamin A for non-breastfed children (Tables 4 and 5).

**Table 4:**
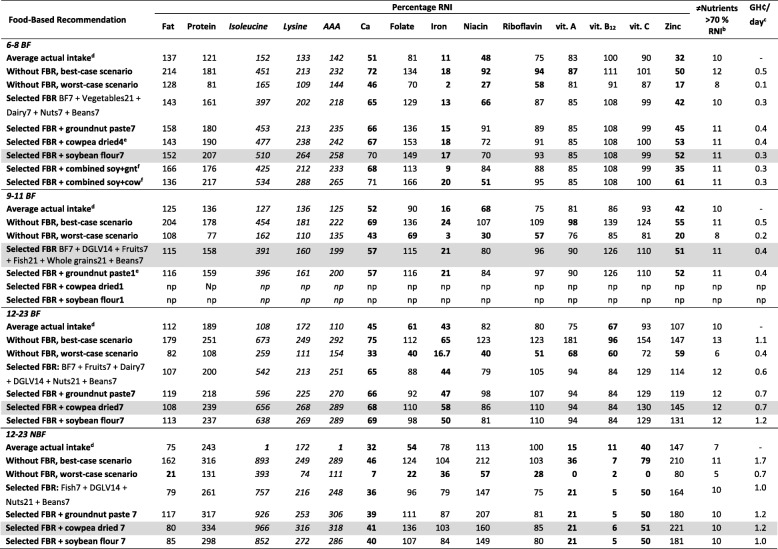
Evaluation of nutrient levels per target group for the minimised diets: the final set of selected food based recommendations (FBRs), the selected FBRs plus 1 serving per day groundnut, the selected FBRs plus 1 serving per day cowpea, the selected FBRs plus 1 serving per day soybean flour and the selected FBRs plus combination of groundnut, cowpea and soybean if possible within energy constraints^a^

6-8 BF = breastfed children of 6-8 months, 9-11 BF = breastfed children of 9-11 months, 12-23 BF = breastfed children of 12-23 months, 12-23 NBF = non-breastfed children of 12-23 months, BF7 = average breastmilk 7 times per week (every day), DGLV = dark green leafy vegetables, Meat = meat, fish or egg food group, Nuts = nuts, seeds and unsweetened products sub food group, soy+gnt = soybean flour plus groundnut paste and soy+cow = soybean flour plus cowpea (tested with servings as stated above in table as individual addition). np = not possible within energy constraints

**Bold values** = Values below 100% RNI for the best-case scenario and values below 70% RNI for the worst-case scenario of the modelled diets and for average actual intake

Grey boxes = for each target group the final set of recommendations that achieved >70% of the RNI in the worst-case scenario for most nutrients but below the 75ht percentile of daily diet cost^a^Values are expressed as percentage of recommended nutrient intakes (RNI)

^b^Total number of nutrients that are above 70% of RNI

^c^Total cost of modelled diet per day in Ghanaian Cedi’s (GH₵)

^d^Average actual intake = percentage RNI covered by average actual intake of target group

^e^Highest servings per week possible within energy constraints

^f^Not possible to combine all three legumes within energy constraints

**Table 5:** Final sets of selected food-based recommendations (in servings per day), including additional extra recommendations for grain legumes for young children per age group and breastfeeding state, and the remaining problem nutrients

Foods	6-8BF	9-11BF	12-23 BF	12-23 NBF
	recommended servings/day			
Breast milk	Every day	Every day	Every day	
Vegetables	3 servings	2 servings of dark green leafy vegetables	2 servings of dark green leafy vegetables	2 servings of dark green leafy vegetables
Dairy	1 serving		1 serving	
Whole grains		3 servings	1 serving	1 serving
Fruits		1 serving	1 serving	
Fish		3 servings		1 serving
Nuts and/or seeds	1 serving		3 servings	3 servings
Beans	1 serving	1 serving	1 servings	1 servings
*Extra cowpea*			*1 serving*	*1 serving*
*Extra soybean*	*1 serving*			
Problem nutrients without addition of extra legumes	calcium, niacin, iron, zinc	calcium, iron, zinc	calcium, iron	calcium, vit. A, vit. B_12_, vit. C
Problem nutrients with addition of extra legumes	iron, zinc	calcium, iron, zinc	calcium, iron	calcium, vit. A, vit. B_12_, vit. C

6-8 BF = breastfed children of 6-8 months, 9-11 BF = breastfed children of 9-11 months, 12-23 BF = breastfed children of 12-23 months, 12-23 NBF = non-breastfed children of 12-23 months

In all four target groups, at least one of the remaining problem nutrients is present in relatively large amounts in cowpea, groundnut and soybean. Groundnut paste, cowpea and soybean flour were added with a frequency of 7 or less to fit within energy constraints, individually and in combination, to the final set of selected FBRs for each target group. For 6-8 BF group, both the addition of four servings of cowpea and the addition of seven servings of soybean per week increased iron and zinc adequacy but not above 70% of RNI in the minimised diets for both nutrients. The addition of seven servings of soybean per week did increase calcium and niacin to 70% of RNI. The combination of adding soybean and cowpea, also increased iron, zinc and calcium adequacy with the latter above 70% of RNI in the minimised diet but niacin decreased to 51% of RNI covered. Addition of combined additional cowpea, groundnut and/or soybean was only possible for this 6-8 BF group, in all other groups the energy limitations were exceeded. For the 9-11 BF group, even the individual addition of legumes was not possible within the energy limitations except for one serving of groundnut paste per week but this did not increase the nutrient adequacy of calcium, iron and zinc above 70% in the final set of selected FBRs. The addition of seven servings of cowpea per week increased calcium and iron adequacy of children 12-23 BF and iron adequacy of 12-23 NBF children but all not above 70% of RNI in the minimised diet (Tables 4 and 5). Comparing minimised diets of the final set of selected FBRs and these FBRs in combination with additional servings of legumes, resulted in the final sets of selected FBRs (Table 6). For all groups problem nutrients remained: for breastfed children calcium, iron and/or zinc and for non-breastfed children calcium, vitamin A, vitamin B12 and vitamin C.

## Discussion

Among IYC in rural Northern Ghana, 40% currently consumed legumes with an average portion size of about 20 g per day contributing more than 10% of their total protein, folate, iron and niacin intake with largest contributions among older children and non-breastfed children (Table 3). The final sets of FBRs that fit within the current dietary patterns included legumes. These FBRs provided adequate protein and EAAs but not of calcium, iron, niacin and/or zinc among breastfed children and of calcium, vitamin C, vitamin B12 and vitamin A among children 12-23 NBF (Table 6). FBRs combined with extra legumes on top of the current dietary pattern but within energy requirements, improved adequacy of calcium, iron, niacin and zinc but only reached sufficient amounts for calcium among 6-8 BF children.

### Legume consumption

Although legume consumption among IYC was relatively common, 40% of our study population consumed no grain legumes while the other 60% consumed only relatively small portion sizes (Table 3). As such, they did not adhere with recommendations promoted by the Ministry of Health in Ghana to consume a cereal-legume complementary food called ‘Weanimix’ but ate instead a cereal based porridge. ‘Weanimix’ contains 75 to 80% maize, 10 to 15% soybean or cowpea and 10% groundnut improving the energy and protein content compared with the use of maize alone [53]. The low legume consumption may have several reasons. A study investigating the acceptability of cowpea by caregivers of schoolchildren in rural Northern Ghana, found that despite cowpea being well accepted in the area, availability on the market, high prices, time required to cook cowpea, post-harvest loss due to insect pests and the resulting short storage time were barriers to give cowpeas to their children [54]. Although almost all caregivers reported that their schoolchildren like to eat cowpea, half of them thought that cowpeas are not easily digested by children and make them feeling uneasy. Caregivers of IYC in Ethiopia also reported to perceive pulses to be not well tolerated and to cause stomach problems in IYC [27]. In addition, our data was collected in the ‘hunger season’ which is the longest period after the previous harvest, and therefore probably most rural households run out of legume stock and indeed found it expensive to buy legumes as prices increase a few months after harvest [55] leading to reduced consumption.

Compared with children not consuming legumes, the intake of most nutrients is greater among children consuming legumes (except for isoleucine and AAA), also of nutrients not present in high concentrations in grain legumes such as vitamin A and vitamin C (Table 3). An explanation for this phenomenon may be related to legumes being regarded as “poor man’s meat” [56], and children from higher socio-economic status may also consume other (more expensive) micronutrient rich foods in addition to legumes. However, we found no differences in socio-economic status indicators between the households of children consuming or not consuming legumes. A more recent study also reported that legumes are consumed across socio-economic strata [54]. A more plausible explanation is that legumes are rarely consumed in isolation, but are often combined in dishes with other micronutrients rich foods such as local vegetables and dried fish. Promoting legume consumption among IYC may therefore also increase consumption of other micronutrient rich foods and improve adequate intake of not only nutrients provided by the legumes.

### Legumes and protein gaps

Among our study population, we found that legumes contributed about 5% to total protein intake among children of below 12 months with a larger contribution among older and non-breastfed children (11% and 22% for children 12-23 BF and 12-23 NBF, respectively) (Table 3). These percentage were larger than observed in diets of rural Ethiopian IYC where legumes contributed less than 4% of total protein intake with no difference according to age. Intake of milk and milk products were high in Ethiopian IYC diets, unlike Ghana, and contributed more to protein intake than legumes [27]. With regard to the group of non-breastfed children we had a limited sample size of 29 children (the vast majority of children of this age were still breastfed) and the foods and portion sizes consumed may not be estimated robustly. However, as we sampled all non-breastfed children we consider our estimates to be realistic. As previously found [22, 27, 57], total protein intake from the cereal based diet appears to be more-or-less sufficient in our study population (only 13% of breastfed and none of the non-breastfed children had a protein intake below their requirements). Nevertheless, the quality of protein intake in terms of EAAs might be at stake, especially in diets of stunted children [19, 20, 26]. Most children in our study, which also had high prevalence of stunted children, had sufficient EAA intake to meet their requirements (Table 2). Previous studies measured EAA intakes of IYC using a metabolomics approach to measure serum amino acids and food balance sheets [19, 20], which might explain the differences compared to our findings. Randomised controlled trials are needed to confirm the relationship between protein quality intake and stunting. In line with our findings, Suri DJ, Tano-Debrah K and Ghosh SA [26] found that a traditional cereal–soybean blend made in Ghana did meet protein quality requirements except for lysine.

We may have underestimated protein and EAA requirements, as well as overestimated their intake. The established EAA requirements might be insufficient for young children in developing countries where energy deficits and infectious diseases are common and catch-up growth is needed [19, 20]. In case of an energy deficit, as is the case among more than 20% in all four target groups, part of the protein intake will be converted and used as energy. A diet that is moderately deficient in energy (5% below requirement) can increase protein needs by 10% [58]. Calculations of protein needs in relation to energy intake depend on many factors such as age, sex and physical activity and more research is needed for estimations of extra requirements in relation to energy deficit [50]. In case of infectious diseases, activation of the immune system may limit EAAs to support growth [59]. The absorption and utilization of amino acids in foods is also important to consider as it decreases the effective protein available in the body [19]. Trypsin in legumes, an anti-nutritive component, for example, reduces protein digestibility up to 50% [60] and we did not correct for protein digestibility in our study. In addition, for the breastfed children in our population it is unsurprising that we found EAAs intake to be sufficient as current EAAs requirements for IYC are based on breastmilk content [50] and we assumed average breastmilk intake [40]. Actual breastmilk intake may be lower than the assumed daily average quantity, especially when meal frequency of complementary feeding increases [46]. Further, EAAs content of breastmilk in rural sub-Saharan Africa may be less than what we assumed based on a recent review with only few studies from Africa with considerably higher concentrations compared to WHO values [41, 50]. Despite our suspicion that we overestimated protein intake as requirements are probably elevated, we did not observe any symptoms of oedema which would indicate protein deficiency.

Among non-breast children, the EAA intakes of isoleucine and AAA did not meet requirements for all children (Table 2). This confirms the benefit of extending breastfeeding also after 1 year of age to cover the EAA requirements [61]. Like animal-source foods, breastmilk is considered to contain good quality protein as it is highly digestible and contains all EAAs in adequate amounts [62]. Therefore we expected to find larger numbers of children not meeting EAAs requirements among older non-breastfed children.

Linear programming also showed that both total protein and EAAs were not problem nutrients in the current diet (when also energy needs are met), nor were isoleucine, lysine and AAA problem nutrients among the non-breastfed group. The developed FBRs, when adopted fully, would ensure a protein and EAAs intake far above the requirements and adding extra legumes was not needed to reach adequacy.

### Legumes and micronutrient gaps

In contrast to protein and EAAs intake, intake of most micronutrients was generally low in all our four target groups including calcium, folate, iron (except for the non-breastfed children), niacin and zinc (Table 2), the nutrients that are relative high in grain legumes and generally found to be deficient in IYC’s diets in developing countries [63, 64]. These findings confirm the need to improve complementary feeding practises [65] for which increasing grain legume intake might be an effective strategy.

Breastfeeding contributed most to all nutrient intakes of children below 12 months (See Additional file 6) but after six months breastmilk alone is not sufficient anymore to cover their nutrient requirements [61]. Given their limited capacity to digest complementary foods [46], additional nutrient-dense foods are needed to cover all micronutrient requirements but these are often lacking [57, 66]. This is especially the case in developing countries as found in our study, due to two main reasons. First, the availability and affordability of nutrient-dense foods is limited. Second, cultural beliefs and practices limit the provision of nutrient-dense foods to the youngest children [67], also in the case of grain legumes [27, 54]. Besides the greatest needs of the youngest children for micronutrient-dense foods, they tend not to eat from the family pot whereas older children do [67]. The family pot is likely to include more nutrient-dense foods compared with foods given to the youngest children. Among non-breastfed children, there is more room in terms of energy for intake of nutrient-dense foods other than breastmilk. Our results suggest that this may have resulted in slightly more sufficient nutrient intakes but only for the nutrients not high in breastmilk such as iron, zinc, folate and niacin [40].

As legumes contain relatively large amounts of micronutrients that are inadequate among the majority of our study population and current intake of legumes is low especially among children of below 12 months, increasing legume consumption may improve micronutrient intakes of all our four target groups. This was confirmed by our final sets of FBRs modelled for all our four target groups that all included the recommendation to consume legumes every day: 1 serving of beans for all four target groups and 3 servings of nuts for children of above 12 months (Table 6). Despite the final FBRs did indeed improve the adequacy of calcium, folate, iron, niacin and zinc intake, these FBRs did not achieve the criteria selected to define a low risk of inadequate intakes for all children in the population in all four target groups except for folate. Other studies that developed FBRs using similar methods, also found that these similar problem nutrients could not be covered within the current dietary pattern of young children and additional interventions are needed [32, 68]. As legumes are relatively high in calcium, iron, niacin and zinc we combined the final sets of FBRs with extra recommendations on legumes on top of their dietary pattern. Again this further improved adequacy of remaining problem nutrients in most cases for all groups but only sufficiently improved calcium and niacin adequacy of 6-8 BF children. Despite the high iron and zinc content of legumes, the bioavailability of these nutrients is weak due to the high content of anti-nutrient components such as phytate that can drastically limiting the uptake of these nutrients [9, 10]. Among children 9-11 BF, the final set of FBRs already covered most of energy needs thereby leaving no room for extra legumes within the energy constraints of the current diet. Modelling FBRs including extra legumes outside of the current dietary pattern from the start may (partly) replace FBR of whole grains and potentially could result in adequate intakes of calcium, iron and/or zinc for this group. Further adding soybean, which contains relatively more calcium than other grain legumes, in higher portion size or frequency to FBRs of children of above 9 months may result in adequate calcium intakes. Nevertheless, as soybean is rarely consumed in Northern Ghana [69] adoption of such a FBR might be challenging.

### Implementation of food-based recommendations

As FBRs are based on the actual dietary patterns and their costs, the foods recommended are assumed to be available, affordable and acceptable for the target population [70]. However, the analysis is based on the distribution of the types and frequencies of foods consumed, and often uses the extremes of these distributions to develop FBRs that cover most nutrient needs. Using these extremes may limit the actual adoption of the FBRs by all IYC, for example, due to beliefs about legume consumption and/or limited availability of legumes in some of the households where probably legume consumption is already low. Therefore before implementation of FBRs, their effectiveness need to be tested, as well as the most effective strategy for behavioural change communication interventions identified [71], and the potential barriers for adoption investigated. Furthermore, the FBRs first need to be aligned across our target groups [47]. An additional serving of fish for 12-23 BF children and additional serving of dairy and nuts for 9-11 BF children would align our FBRs for IYC. Nevertheless, adding dairy and nuts to FBRs for 9-11 BF was not possible within energy constraints.

## Conclusions

This study showed that current grain legume intake among rural Ghanaian IYC contributes to nutrient intakes especially protein, folate, iron and niacin but in insufficient quantities to reach adequacy of all nutrients. Both current protein and EAAs intake were adequate in our study population making increasing grain legume consumption within the dietary pattern of IYC in rural Ghana unnecessary. Therefore increased consumption of legumes was not needed to improve protein intake. By contrast intake of most micronutrients was low in our study population, and increasing legume consumption within the dietary pattern of IYC in rural Ghana does have potential to increase adequacy of micronutrients. Nevertheless, consumption of additional legume foods resulted in only slight improvements in micronutrient adequacy on top of the current dietary patterns. Therefore other interventions are also needed such as other food-based approaches for example increasing the availability and accessibility of micronutrient-dense foods and/or fortification or supplementation strategies to improve micronutrient adequacy of infants and young children in rural Ghana.

## Additional files

Additional file 1: Energy, fat, protein and essential amino acid requirements used for calculating percent of children with nutrient intakes below requirements, based on reference weight and actual weight. (DOCX 18 kb)

Additional file 2: Micronutrient requirements used for calculating percent of children with nutrient intakes below requirements. (DOCX 18 kb)

Additional file 3: Distribution of daily diet costs^a^ per target group. (DOCX 16 kb)

Additional file 4 All non-condiment foods consumed by >5% of target children with a median portion size of at least 1 gram in Karaga district, median serving size (g/day) and percentage of children consuming each food. (DOCX 20 kb)

Additional file 5: Dietary pattern with minimum and maximum servings per week by target group. (DOCX 20 kb)

Additional file 6: The count of nutrients that foods contributed >5% to specific nutrient intake in the best optimised diet, for each age group (out of 14 nutrients). (DOCX 17 kb)

Additional file 7: Maximum percentage of RNI covered in the maximised diets, without FBR constraints. (DOCX 17 kb)

## Abbreviations

12-23 BF: 12-23 months breastfed children
12-23 NBF: 12-23 months non-breastfed children
6-8 BF: 6-8 months breastfed children
9-11 BF: 9-11 months breastfed children
AAA: Aromatic amino acids
ADMR: Acceptable macronutrient distribution range
EAAs: Essential amino acids
EARS: Estimated average requirements
FAO: Food and Agriculture Organisation of the United States
FBRs: Food-based recommendations
FCT: Food composition table
IYC: infants and young children
iZiNCG: International Zinc Nutrition Consultative Group
RAE: retinol activity equivalent
RNIs: Recommended nutrient intakes
SAA: Sulphur-containing amino acids
UNU: United Nations University
WHO: World Health Organisation

## Footnotes

1 Grain legumes are crops of the legume family (Fabaceae) cultivated specifically for their seeds for human food and animal feed. The most commonly grown grain legumes in West Africa are cowpea and groundnut, although soybean is increasing in popularity. The leaves of some grain legumes (e.g. cowpea) are also consumed by humans.
