# Supplementary material for: Current and potential role of grain legumes on protein and micronutrient adequacy of the diet of rural Ghanaian infants and young children: using linear programming
Source: Nutr J. 2019 Feb 21;18:12. doi: 10.1186/s12937-019-0435-5 (PMC6385461; doi:10.1186/s12937-019-0435-5)
Supplement: Supplementary file 1 — Energy, fat, protein and essential amino acid requirements used for calculating percent of children with nutrient intakes below requirements, based on reference weight and actual weight. (DOCX 18 kb) [file 12937_2019_435_MOESM1_ESM.docx]

**Additional file A.** Energy, fat, protein and essential amino acid requirements used for calculating percent of children with nutrient intakes below requirements, based on reference weight and actual weight

|  | **children 6-8 mo** | | **children 9-11 mo** | | **children 12-23 mo** | | |
| --- | --- | --- | --- | --- | --- | --- | --- |
|  | **Reference weight**  (7.98 kg) | **Actual weight**  (7.03 kg) | **Reference weight**  (9.03kg) | **Actual weight**  (7.54kg) | **Reference weight**  (10.74kg) | **Actual weight BF**  (8.48kg) | **Actual weight NBF**  (9.43kg) |
| Energy (kcal)^a^ | **614** | **541** | **695** | **581** | **886** | **700** | **778** |
| Fat (g)^b^ | **20.5** | 18.0 | **23.2** | 19.4 | **29.5** | 23.3 | 25.9 |
| Protein (g)^c^ | **9.1** | 8.0 | **10.3** | 8.6 | **11.1** | 8.7 | 9.7 |
| *Histidine^d^ (mg)^d^* | ***182*** | 160 | ***196*** | 163 | ***199*** | 157 | 175 |
| *Isoleucine^d^ (mg)^d^* | ***291*** | 256 | ***324*** | 271 | ***343*** | 270 | 301 |
| *Leucine^d^ (mg)^d^* | ***601*** | 528 | ***664*** | 555 | ***697*** | 548 | 611 |
| *Lysine^d^ (mg)^d^* | ***519*** | 456 | ***561*** | 469 | ***575*** | 452 | 504 |
| *SAA (mg)^d^* | ***255*** | 224 | ***278*** | 232 | ***288*** | 226 | 252 |
| *AAA (mg)^d^* | ***473*** | 416 | ***504*** | 421 | ***509*** | 400 | 446 |
| *Threonine^d^ (mg)^d^* | ***282*** | 248 | ***298*** | 249 | ***299*** | 235 | 262 |
| *Tryptophan^d^ (mg)^d^* | ***77*** | 68 | ***82*** | 68 | ***82*** | 64 | 72 |
| *Valine^d^ ^d^(mg)^d^* | ***391*** | 344 | ***437*** | 366 | ***465*** | 365 | 407 |

SAA = sulphur-containing amino acids (methionine and cystine); AAA = aromatic amino acids (phenylalanine and tyrosine). **Bold values** = are values used for calculating percent of children with nutrient intakes below requirements (in Table 2).  ^a^based on average reference or actual body weight for age group and algorithm for estimating energy requirements (kcal/kg) (FAO, 2004)
^b^based on the acceptable macronutrient distribution range (ADMR) of 30% of daily energy requirements (FAO, 2010)
^c^based on average reference body weight for age group and algorithm for estimating protein requirement (g/kg), safe intakes (FAO, 2007)
^d^based on daily total protein requirements and algorithms for each essential amino acid requirements (mg/g protein) using safe intakes for 0.5 year old children 6-8mo group, average of safe intakes for 0.5 years and 1 to 2 years old children for 9-11mo group and safe intakes for 1 to 2 years old children for 12-23mo group (WHO, 2007).
