# Supplementary material for: Current and potential role of grain legumes on protein and micronutrient adequacy of the diet of rural Ghanaian infants and young children: using linear programming
Source: Nutr J. 2019 Feb 21;18:12. doi: 10.1186/s12937-019-0435-5 (PMC6385461; doi:10.1186/s12937-019-0435-5)
Supplement: Supplementary file 2 — Micronutrient requirements used for calculating percent of children with nutrient intakes below requirements. (DOCX 18 kb) [file 12937_2019_435_MOESM2_ESM.docx]

**Additional file B.** Micronutrient requirements used for calculating percent of children with nutrient intakes below requirements

|  | **children 6-8 mo** | | **children 9-11 mo** | | **children 12-23 mo** | | |
| --- | --- | --- | --- | --- | --- | --- | --- |
| *Micronutrients* | *RNI^a^* | *EAR^b^*  *CV* | *RNI^a^* | *EAR^b^* | *RNI^a^* | *EAR^c^* | *CV* |
| Calcium (mg) | 400 | n/a | 400 | n/a | 500 | **417** | 1.2 |
| Folate (μg DFE) | 80 | **65** | 80 | **65** | 150 | **120** | 1.25 |
| Iron (mg)^d^ | 18.6 | **6.9** | 18.6 | **6.9** | 11.6 | **3.0** | n/a |
| Niacin (mg) | 4 | n/a | 4 | n/a | 6 | **4.6** | 1.3 |
| Riboflavin (mg) | 0.4 | n/a | 0.4 | n/a | 0.5 | **0.4** | 1.25 |
| Thiamine (mg) | 0.3 | n/a | 0.3 | n/a | 0.5 | **0.4** | 1.25 |
| Vitamin A (μg RAE) | 400 | n/a | 400 | n/a | 400 | **286** | 1.4 |
| Vitamin B_6_ (mg) | 0.3 | n/a | 0.3 | n/a | 0.5 | **0.4** | 1.25 |
| Vitamin B_12_ (μg) | 0.7 | n/a | 0.7 | n/a | 0.9 | **0.7** | 1.3 |
| Vitamin C (mg) | 30 | n/a | 30 | n/a | 30 | **25** | 1.2 |
| Zinc (mg)^e^ | 5 | **4** | 5 | **4** | 3 | **2** | 1.2 |

SAA = sulphur-containing amino acids (methionine and cystine); AAA = aromatic amino acids (phenylalanine and tyrosine); CV = conversion factor. **Bold values** = are values used for calculating percent of children with nutrient intakes below requirements (in Table 2). ^a^RNIs from FAO/WHO (2004) except for zinc based on RNI from iZiNCG (2004)
^b^No conversion factors available for children below 12 months old, except for folate EAR from FAO/WHO (2004), for iron EAR from IOM (2001) and for zinc EAR from iZiNCG (2004). ^c^EARs calculated from RNIs (FAO/WHO 2004), using conversion factors (Allen, et al., 2006) except for iron EAR from IOM (2001) and for zinc EAR from iZiNCG (2004).
^d^Assuming 5% bioavailability.
^e^Assuming unrefined cereal-based diets.
