# Supplementary material for: Current and potential role of grain legumes on protein and micronutrient adequacy of the diet of rural Ghanaian infants and young children: using linear programming
Source: Nutr J. 2019 Feb 21;18:12. doi: 10.1186/s12937-019-0435-5 (PMC6385461; doi:10.1186/s12937-019-0435-5)
Supplement: Supplementary file 3 — Distribution of daily diet costsa per target group. (DOCX 16 kb) [file 12937_2019_435_MOESM3_ESM.docx]

**Additional file D.** Distribution of daily diet costs^a^ per target group

| **Target group** | **25th** | **50th** | **75th** |
| --- | --- | --- | --- |
| 6 to 8 months BF | 0.08 | 0.18 | **0.39** |
| 9 to 11 months BF | 0.16 | 0.34 | **0.71** |
| 12 to 23 months BF | 0.45 | 0.77 | **1.23** |
| 12 to 23 months NBF | 0.99 | 1.51 | **2.29** |

^a^Daily diet cost per child were calculated by summing the price of each quantity of a food consumed per child, using the average price per edible 100 g portion (prices were collected from three different food sellers in the area).
