# Supplementary material for: Current and potential role of grain legumes on protein and micronutrient adequacy of the diet of rural Ghanaian infants and young children: using linear programming
Source: Nutr J. 2019 Feb 21;18:12. doi: 10.1186/s12937-019-0435-5 (PMC6385461; doi:10.1186/s12937-019-0435-5)
Supplement: Supplementary file 4 — All non-condiment foods consumed by >5% of target children with a median portion size of at least 1 gram in Karaga district, median serving size (g/day) and percentage of children consuming each food. (DOCX 20 kb) [file 12937_2019_435_MOESM4_ESM.docx]

**Additional file C.** All non-condiment foods consumed by >5% of target children with a median portion size of at least 1 gram in Karaga district, median serving size (g/day) and percentage of children consuming each food

|  | **6-8 BF**  *n=97* | | **9-11 BF**  *n=97* | | **12-23 BF**  *n=114* | | **12-23 NBF**  *n=29* | |
| --- | --- | --- | --- | --- | --- | --- | --- | --- |
| **Foods** | Median amount in g/day (% children consumed), *min - max frequency* | | | | | | | |
| ***Added fats*** |  | | | | | | | |
| Oil palm |  | |  | | 5 (7) | *0 - 7* |  | |
| Oil vegetable frytol |  | | 3 (14) | *0 - 7* | 6 (17) | *0 - 7* | 6 (21) | *0 - 7* |
| Vegetable Oil | 14 (11) | *0 - 7* | 15 (31) | *0 - 7* | 13 (52) | *0 - 7* | 17 (48) | *0 - 7* |
| ***Added sugar*** |  | | | | | | | |
| Sugar white refined | 6 (52) | *0 - 7* | 9 (52) | *0 - 7* | 14 (59) | *0 - 7* | 16 (66) | *0 - 7* |
| ***Bakery & breakfast cereals*** |  | | | | | | | |
| Biscuit sweet | 9 (6) | *0 - 7* |  | |  | |  | |
| Bread sugar |  | |  | | 50 (6) | *0 - 7* | 73 (21) | *0 - 7* |
| ***Beverages*** |  | |  |  |  | |  | |
| Creamer non diary powder |  | | 2 (8) | *0 - 7* | 7 (12) | *0 - 7* | 6 (24) | *0 - 7* |
| ***Dairy products*** |  | | | | | | | |
| Milk cow powder skimmed | 3 (7) | *0 - 7* | 2 (6) | *0 - 7* | 5 (7) | *0 - 7* |  |  |
| ***Fruits*** |  | | | | | | | |
| Melon water raw |  | | 72 (5) | *0 - 7* | 126 (7) | *0 - 7* |  | |
| ***Grains & grain products*** |  | | | | | | | |
| Guinea corn dough whole grain RT^3^ boiled | 20 (17) | *0 - 3* | 22 (20) | *0 - 3* | 48 (15) | *1 - 2* | 66 (17) | *1 - 7* |
| Guinea corn flour whole grain RT boiled | 40 (19) | *0 - 3* | 33 (20) | *0 - 3* | 27 (15) | *1 - 2* |  | |
| Maize dough whole grain white RT boiled | 27 (44) | *0 - 7* | 36 (43) | *0 - 7* | 50 (37) | *1 - 7* | 38 (31) | *1 - 7* |
| Maize flour whole grain white RT boiled | 40 (30) | *0 - 6* | 38 (55) | *0 - 7* | 65 (76) | *2 - 7* | 123 (90) | *3 - 7* |
| Maize grain dried white RT boiled |  | | 11 (8) | *0 - 1* | 53 (10) | *0 - 2* | 11 (21) | *1 - 7* |
| Millet dough whole grain RT boiled |  | | 10 (6) | *0 - 1* | 33 (9) | *0 - 1* |  | |
| Millet flour whole grain RT boiled |  | | 13 (8) | *0 - 1* | 15 (8) | *0 - 1* |  | |
| Noodles instant RT boiled |  | | 3 (7) | *0 - 4* | 9 (6) | *0 - 7* |  | |
| Rice local brown unpolished raw RT boiled | 40 (11) | *0 - 2* | 21 (29) | *0 - 4* | 56 (49) | *2 - 6* | 103 (48) | *2 - 7* |
| Rice white polished raw RT boiled |  | | 41 (5) | *0 - 3* |  | |  | |
| ***Legumes, nuts & seeds*** |  | | | | | | | |
| Beans soya dried raw RT boiled |  | |  | | 1 (7) | *0 - 7* |  | |
| Cowpea white dried whole RT boiled | 24 (8) | *0 - 7* | 10 (11) | *0 - 7* | 23 (17) | *0 - 4* | 42 (24) | *0 - 7* |
| Groundnut flour with fat RT boiled |  |  | 4 (9) | *0 - 7* | 5 (11) | *0 - 7* | 13 (21) | *0 - 4* |
| Groundnut roasted paste RT boiled | 7 (6) | *0 - 7* | 4 (21) | *0 - 7* | 8 (44) | *0 - 7* | 25 (45) | *0 - 7* |
| Groundnut shelled dried raw RT boiled |  | |  | |  | | 1 (76) | *0 - 7* |
| Neri roasted RT boiled |  | |  | | 4 (8) | *0 - 7* | 15 (24) | *0 - 3* |
| Pigeon peas dried RT boiled |  | |  | | 23 (9) | *0 - 2* |  | |
| ***Meat, fish & eggs*** |  | | | | | | | |
| Fish anchovies smoked dried RT boiled | 1 (28) | *0 - 7* | 2 (56) | *0 - 7* | 2 (84) | *0 - 7* | 4 (83) | *0 - 7* |
| Fish herrings smoked dried RT boiled |  | | 1 (5) | *0 - 7* |  | |  | |
| Mackerel canned in tomato sauce RT boiled |  | | 1 (6) | *0 - 7* | 1 (7) | *0 - 7* |  | |
| ***Starchy roots & other starchy plant foods*** |  | | | | | | | |
| Cassava dough roasted |  | | 3 (5) | *0 - 7* | 8 (7) | *0 - 3* |  | |
| Cassava flour RT boiled | 3 (7) | *0 - 7* | 9 (8) | *0 - 4* | 9 (9) | *0 - 4* |  | |
| ***Vegetables*** |  | | | | | | | |
| Ayoyo leaves raw RT boiled | 5 (14) | *0 - 5* | 3 (29) | *0 - 7* | 7 (45) | *0 - 7* | 18 (31) | *0 - 7* |
| Bra leaves raw RT boiled | 7 (7) | *0 - 2* | 8 (17) | *0 - 7* | 17 (27) | *0 - 7* | 19 (59) | *0 - 7* |
| Okro fruit dried powder RT boiled |  | | 1 (7) | *0 - 1* | 3 (14) | *0 - 7* | 4 (28) | *0 - 7* |
| Okro fruit raw RT boiled |  | | 12 (14) | *0 - 7* | 25 (11) | *0 - 7* | 27 (24) | *0 - 7* |
| Onion bulb raw RT boiled | 2 (9) | *0 - 7* | 2 (32) | *0 - 6* | 2 (50) | *0 - 7* | 6 (31) | *0 - 7* |
| Tomato paste concentrated RT boiled | 4 (7) | *0 - 7* | 3 (24) | *0 - 7* | 5 (37) | *0 - 7* | 10 (28) | *0 - 7* |
| ***Breastmilk*** | *660* | *7 - 7* | *616* | *7 - 7* | *549* | *7 - 7* |  | |

6-8 BF = breastfed children of 6-8 months, 9-11 BF = breastfed children of 9-11 months, 12-23 BF = breastfed children of 12-23 months, 12-23 NBF = non-breastfed children of 12-23 months.
