# Supplementary material for: Current and potential role of grain legumes on protein and micronutrient adequacy of the diet of rural Ghanaian infants and young children: using linear programming
Source: Nutr J. 2019 Feb 21;18:12. doi: 10.1186/s12937-019-0435-5 (PMC6385461; doi:10.1186/s12937-019-0435-5)
Supplement: Supplementary file 5 — Dietary pattern with minimum and maximum servings per week by target group. (DOCX 20 kb) [file 12937_2019_435_MOESM5_ESM.docx]

**Additional file E.** Dietary pattern with minimum and maximum servings per week by target group

|  | **6-8 BF**  *n=97* | | **9-11 BF**  *n=97* | | **12-23 BF**  *n=114* | | **12-23 NBF**  *n=29* | |
| --- | --- | --- | --- | --- | --- | --- | --- | --- |
| **Food groups & Sub food groups^3^** | **Servings per week** | | | | | | | |
|  | Min^4^ | Max^5^ | Min | Max | Min | Max | Min | Max |
| **Grains & grain products** | **0** | **21** | **0** | **28** | **7** | **28** | **7** | **35** |
| Whole grains and products unenriched/unfortified | 0 | 21 | 0 | 28 | 7 | 28 | 7 | 35 |
| Refined grains and products unenriched/unfortified | - | - | 0 | 7 | 0 | 7 | - | - |
| **Starchy roots & other starchy plant foods** | **0** | **7** | **0** | **7** | **0** | **7** | **-** | **-** |
| Other starchy plant foods | 0 | 7 | 0 | 7 | 0 | 7 | - | - |
| **Legumes, nuts & seeds** | **0** | **21** | **0** | **28** | **0** | **28** | **7** | **28** |
| Cooked beans, lentils, peas | 0 | 7 | 0 | 7 | 0 | 7 | 0 | 7 |
| Nuts, seeds, and unsweetened products | 0 | 14 | 0 | 21 | 0 | 21 | 0 | 21 |
| Soybeans and products | - | - | - | - | 0 | 7 | - | - |
| **Meat, fish & eggs** | **0** | **7** | **0** | **21** | **0** | **14** | **0** | **14** |
| Small, whole fish, with bones | 0 | 7 | 0 | 21 | 0 | 14 | 0 | 14 |
| **Beverages (non-dairy or blended dairy)** | **-** | **-** | **0** | **7** | **0** | **7** | **0** | **14** |
| Other beverages | - | - | 0 | 7 | 0 | 7 | 0 | 7 |
| **Dairy products** | **0** | **7** | **0** | **7** | **0** | **7** | **-** | **-** |
| Fluid or powdered milk (fortified) | 0 | 7 | 0 | 7 | 0 | 7 | - | - |
| **Vegetables** | **0** | **21** | **0** | **28** | **0** | **28** | **7** | **35** |
| Vitamin A source dark green leafy vegetables | 0 | 7 | 0 | 14 | 0 | 14 | 0 | 14 |
| Vitamin A source other vegetables | 0 | 7 | 0 | 7 | 0 | 7 | 0 | 7 |
| Other vegetables | 0 | 7 | 0 | 7 | 0 | 14 | 0 | 14 |
| Vitamin C-rich vegetables | - | - | 0 | 7 | 0 | 7 | 0 | 7 |
| **Fruits** | **-** | **-** | **0** | **7** | **0** | **7** | **-** | **-** |
| Other fruit | - | - | 0 | 7 | 0 | 7 | - | - |
| **Bakery & breakfast cereals** | **0** | **7** | **-** | **-** | **0** | **7** | **0** | **7** |
| Sweetened bakery products unenriched/unfortified | 0 | 7 | - | - | 0 | 7 | 0 | 7 |
| **Added fats** | **0** | **7** | **0** | **14** | **0** | **14** | **0** | **14** |
| Vegetable oil (unfortified) | 0 | 7 | 0 | 7 | 0 | 7 | 0 | 7 |
| Vegetable oil (fortified) | - | - | 0 | 7 | 0 | 7 | 0 | 7 |
| Red palm oil | - | - | - | - | 0 | 7 | - | - |
| **Added sugars** | **0** | **7** | **0** | **7** | **0** | **7** | **0** | **7** |
| Sugar (non-fortified) | 0 | 7 | 0 | 7 | 0 | 7 | 0 | 7 |
| ***Breastmilk*** | *6.9* | *7* | *6.9* | *7* | *6.9* | *7* | *-* | *-* |

6-8 BF = breastfed children of 6-8 months, 9-11 BF = breastfed children of 9-11 months, 12-23 BF = breastfed children of 12-23 months, 12-23 NBF = non-breastfed children of 12-23 months.

*^3^Food groups and sub food groups are classified as in Optifood ^4^5^th^ percentile of the weekly frequency was used, ^5^95^th^ percentile of the weekly frequency was used*
