# Supplementary material for: Current and potential role of grain legumes on protein and micronutrient adequacy of the diet of rural Ghanaian infants and young children: using linear programming
Source: Nutr J. 2019 Feb 21;18:12. doi: 10.1186/s12937-019-0435-5 (PMC6385461; doi:10.1186/s12937-019-0435-5)
Supplement: Supplementary file 6 — The count of nutrients that foods contributed >5% to specific nutrient intake in the best optimised diet, for each age group (out of 14 nutrients). (DOCX 17 kb) [file 12937_2019_435_MOESM6_ESM.docx]

**Additional file F.** The count of nutrients that foods contributed >5% to specific nutrient intake in the best optimised diet, for each age group (out of 14 nutrients)

| **Foods** | 6-8mo BF | 9-11mo BF | 12-23mo BF | 12-23mo NBF |
| --- | --- | --- | --- | --- |
| ***Grains*** |  |  |  |  |
| Guinea corn dough |  | 2 |  | 9 |
| Guinea corn flour |  | 6 |  |  |
| Maize flour whole grain white |  |  | 8 | 11 |
| Millet flour whole grain |  | 1 |  |  |
| Rice local brown unpolished raw | 5 |  | 4 | 7 |
| ***Legumes, nuts & seeds*** |  |  |  |  |
| Cowpea white dried whole | 10 | 7 | 7 | 11 |
| Groundnut roasted paste | 9 | 4 | 6 | 11 |
| Groundnut flour with fat |  |  | 4 | 1 |
| Neri roasted |  |  | 2 | 5 |
| Pigeon peas dried |  |  | 3 |  |
| ***Vegetables*** |  |  |  |  |
| Ayoyo leaves raw | 2 |  | 3 | 6 |
| Bra leaves raw |  | 5 | 6 | 6 |
| Okro fruit raw boiled |  | 2 | 3 | 3 |
| Tomato paste concentrated |  |  |  | 2 |
| ***Meat, fish & eggs*** |  |  |  |  |
| Fish anchovies smoked dried |  | 1 | 1 | 2 |
| Fish herrings smoked dried |  | 1 |  |  |
| Mackerel canned in tomato sauce |  | 1 | 1 |  |
| ***Beverages (non-dairy)*** |  |  |  |  |
| Milk cow powder skimmed | 8 | 5 | 8 |  |
| ***Others (fats, fruits)*** |  |  |  |  |
| Oil vegetable Frytol |  | 2 |  | 3 |
| Melon water raw |  |  | 4 |  |
| ***Breastmilk*** | *14* | *14* | *13* | *-* |

6-8 BF = breastfed children of 6-8 months, 9-11 BF = breastfed children of 9-11 months, 12-23 BF = breastfed children of

12-23 months, 12-23 NBF = non-breastfed children of 12-23 months.
