# Supplementary material for: Current and potential role of grain legumes on protein and micronutrient adequacy of the diet of rural Ghanaian infants and young children: using linear programming
Source: Nutr J. 2019 Feb 21;18:12. doi: 10.1186/s12937-019-0435-5 (PMC6385461; doi:10.1186/s12937-019-0435-5)
Supplement: Supplementary file 7 — Maximum percentage of RNI covered in the maximised diets, without FBR constraints. (DOCX 17 kb) [file 12937_2019_435_MOESM7_ESM.docx]

**Additional file G.** Maximum percentage of RNI covered in the maximised diets, without FBR constraints

| **Nutrients** | **6-8 BF** | **9-11 BF** | **12-23 BF** | **12-23 NBF** |
| --- | --- | --- | --- | --- |
| Energy | 174.6 | 165.9 | 186.3 | 223.6 |
| Fat | 213.7 | 204.8 | 179.3 | 315.5 |
| Protein | 181.1 | 177.6 | 251.3 | 161.9 |
| *Isoleucine* | *451.3* | *453.5* | *673.2* | *892.9* |
| *AAA* | *232.1* | *221.7* | *292* | *289.3* |
| *Lysine* | *212.5* | *181.2* | *249.2* | *248.8* |
| Calcium | 71.8 | 68.7 | 74.9 | 45.5 |
| Vitamin C | 100.9 | 123.6 | 154.2 | 78.9 |
| Riboflavin | 94.4 | 109.3 | 123.4 | 102.7 |
| Niacin | 92.4 | 107.4 | 123.4 | 211.8 |
| Folate | 134.4 | 135.5 | 111.6 | 124.2 |
| Vitamin B12 | 110.6 | 138.8 | 96.2 | 6.8 |
| Vitamin A | 86.6 | 97.8 | 180.5 | 36.1 |
| Iron | 18.1 | 23.9 | 65.3 | 103.6 |
| Zinc | 50.0 | 54.5 | 147.4 | 210.2 |

6-8 BF = breastfed children of 6-8 months, 9-11 BF = breastfed children of 9-11 months, 12-23 BF = breastfed children of

12-23 months, 12-23 NBF = non-breastfed children of 12-23 months.
AAA = aromatic amino acids (phenylalanine and tyrosine).

*Grey boxes = problem nutrients, nutrients below 100% RNI in best-case scenario: not possible to meet by any combination of local foods.*
